# Identification of the feline foamy virus Bet domain essential for APOBEC3 counteraction

**DOI:** 10.1186/1742-4690-10-76

**Published:** 2013-07-24

**Authors:** Dragana Slavkovic Lukic, Agnes Hotz-Wagenblatt, Janet Lei, Ann-Mareen Räthe, Michael Mühle, Joachim Denner, Carsten Münk, Martin Löchelt

**Affiliations:** 1 Research Program Infection and Cancer, Department Genome Modifications and Carcinogenesis, German Cancer Research Center (DKFZ), Heidelberg, Germany; 2 Core Facility Genomics and Proteomics, German Cancer Research Center (DKFZ), Heidelberg, Germany; 3 Robert Koch-Institute, Center for HIV and Retroviruses, Berlin, Germany; 4 Clinic for Gasteroenterology, Hepatology and Infectology, Medical Faculty, Heinrich-Heine-University, Düsseldorf, Germany

**Keywords:** APOBEC3, Retrovirus, Foamy virus, Antiviral restriction factors, Bet protein, Host-virus interaction, Virus defence protein

## Abstract

**Background:**

APOBEC3 (A3) proteins restrict viral replication by cytidine deamination of viral DNA genomes and impairing reverse transcription and integration. To escape this restriction, lentiviruses have evolved the viral infectivity factor (Vif), which binds A3 proteins and targets them for proteolytic degradation. In contrast, foamy viruses (FVs) encode Bet proteins that allow replication in the presence of A3, apparently by A3 binding and/or sequestration, thus preventing A3 packaging into virions and subsequent restriction. Due to a long-lasting FV-host coevolution, Bet proteins mainly counteract restriction by A3s from their cognate or highly related host species.

**Results:**

Through bioinformatics, we identified conserved motifs in Bet, all localized in the *bel2* exon. In line with the localization of these conserved motifs within *bel2*, this part of feline FV (FFV) Bet has been shown to be essential for feline A3 (feA3) inactivation and feA3 protein binding. To study the function of the Bet motifs in detail, we analyzed the ability of targeted deletion, substitution, and chimeric FFV-PFV (prototype FV) Bet mutants to physically bind and/or inactivate feA3. Binding of Bet to feA3Z2b is sensitive to mutations in the first three conserved motifs and N- and C-terminal deletions and substitutions across almost the complete *bel2* coding sequence. In contrast, the Bel1 (also designated Tas) domain of Bet is dispensable for basal feA3Z2b inactivation and binding but mainly increases the steady state level of Bet. Studies with PFV Bel1 and full-length FFV Bel2 chimeras confirmed the importance of Bel2 for A3 inactivation indicating that Bel1 is dispensable for basal feA3Z2b inactivation and binding but increases Bet stability. Moreover, the *bel1/tas* exon may be required for expression of a fully functional Bet protein from a spliced transcript.

**Conclusions:**

We show that the Bel2 domain of FV Bet is essential for the inactivation of APOBEC3 cytidine deaminase restriction factors. The Bel1/Tas domain increases protein stability and can be exchanged by related sequence. Since feA3 binding and inactivation by Bet are highly correlated, the data support the view that FV Bet prevents A3-mediated restriction of viral replication by creating strong complexes with these proteins.

## Background

Cellular restriction factors are key players of intrinsic immunity, which acts against viruses immediately after infection [1]. Restriction factors are constitutively expressed in cells but their expression can be increased by interferons produced upon viral infection [2-7]. So far, four anti-retroviral restriction factors have been functionally characterized in detail: APOBEC3, Trim5α, tetherin and SAMHD1 [8-12]. In addition, several other restriction factors have been detected by genome-wide screens but require additional experimental characterization [13,14]. Restriction factors interfere with defined steps in the viral life cycle, leading to attenuation or complete suppression of virus replication. By coevolution with host-encoded restriction factors, current viruses have achieved mechanisms to circumvent this inhibitory activity. Some viruses, for instance, have acquired special proteins that directly counteract restriction factors [1,2,8,12,15]. Due to the interaction of viral proteins with restriction factors, both viral and host-encoded proteins are under constant positive selection to evade or strengthen, respectively, this functional interaction [16]. Host-virus coevolution directly impacts the species-specificity of a given pathogen and is considered as one of the main factors preventing interspecies virus transmission [2].

APOBEC3 (A3) cytidine deaminases are potent antiviral restriction factors. These host proteins deaminate cytidine residues in single-stranded (ss) DNA intermediates generated during retroviral reverse transcription, creating hypermutated genomes with uridine residues [17]. Although considered to deaminate only ssDNA, A3 proteins are also active against double-stranded DNA viruses such as papillomaviruses, probably due to a transient exposure of ssDNA during replication and/or transcription [18]. In addition, it has been shown that A3s deaminate genomes of hepadnaviruses, endogenous retroviruses, retroid elements and possibly even cellular genes [8,19-22]. To restrict retroviral replication, A3 restriction factors must be incorporated into viral particles to edit the forming proviral DNA in the newly infected cell [23]. In contrast, A3s deaminate foamy virus (FV) genomes not only in newly infected cells, but also in virus-producing cells [15] as reverse transcription of FVs may already occur in virus-producing cells [24]. In addition, it has been shown that some human A3s have deaminase-independent activities such as blocking reverse transcription and integration [25-31]. In fact, A3 proteins with mutated catalytic sites have been shown to be active against HIV-1 [25,31,32].

The dynamic coevolution of host defences and virus counter-defence has resulted in a significant expansion of the A3 locus in higher mammals by distinct gene duplication events. In humans, this has led to seven distinct genes/proteins (A3A, B, C, DE, F, G, H), while only four single-domain A3 genes are present in cats [33-37]. Additional complexity of the A3 repertoire in cats is achieved by alternative and complex splicing events of these genes leading to one two-domain feline A3 (feA3) proteins; for details, see refs. [33,37,38].

Two classes of retroviral counter-defence proteins against A3s have been described so far: lentivirus Vif and FV Bet. Data on how other retroviruses replicate in the presence of A3 restriction factors is scarce, but indicate that these viruses have developed other means to avoid A3 packaging [27,39].

The mechanism of A3 inactivation by lentiviral (HIV) Vif is well characterized. Vif acts as an adaptor protein that binds to both A3 and ubiquitin ligase complexes consisting of cullin-5, RING-box 1 and elongins B and C [40]. Thus, Vif induces the ubiquitination and subsequent degradation of A3 by the proteasome [40]. It has been shown that N-terminal regions of Vif bind to huA3G and huA3F while a SOCS box motif mediates ubiquitin ligase complex binding [40,41]. Binding of Vif to A3G is species-specific and substitution of only a single amino acid in human A3G results in a mutant that is no longer targeted by HIV Vif [42].

The FV *bet* genes are expressed by all known exogenous FVs but are also present in the sloth endogenous FV (SloEFV) genome, which is at least 100 million years old [43]. Feline and prototype/primate/human FV (PFV) Bet have been recently shown to counteract defined A3 proteins of feline and human/non-human primate (NHP) origin, resp., and protect FV replication in A3-positive cells [15,44,45]. There is no sequence homology between Bet and Vif and Bet does not contain the SOCS box motif required for E3 ubiquitin ligase complex interactions [46]. In contrast to lentiviral Vif, Bet does not induce A3 degradation [15,44,47]. Bet is thought to directly bind and possibly sequester A3 proteins, preventing their incorporation into viral particles [15,44]. In line with this, Bet is expressed at high levels in infected cells and animals [48] which may be a prerequisite for such a stoichiometric reaction.

Bet is an accessory protein of FVs, viruses that display a complex genetic organization. Together with the essential Bel1/Tas transactivator of both FV promoters, Bet is mainly expressed from the internal promoter located in the 3′end of *env*[49]. Bet is the product of a splicing event that fuses the 5′ domain of *bel1* to the complete *bel2* open reading frame (ORF). All known FVs encode *bet* and, as shown by genome localization, corresponding *bet* genes are also present in endogenous FVs [43,50]. A protein consisting of *bel2* only is not expressed *in vitro*; whether it is expressed *in vivo* is still unknown.

Although it has been shown that FFV Bet binds to all known feA3 proteins, the amino acid residues involved in binding have yet to be identified. It has been shown, however, that FFV Bet-MCS, with a mutation in the Bel2 domain of Bet, is incapable of counteracting feA3 and cannot replicate in A3-positive CrFK cells [15,47,51]. In this FFV proviral genome, a multiple cloning site (MCS) had been introduced inside the *bel2* ORF, leading to the alteration of E^117^L^118^L^119^ residues to ASVRRGP [51]. Despite complete sequence conservation in the rest of Bet, Bet-MCS does not bind or inactivate feA3s, indicating that the mutated region is important for A3 counteraction [15,47]. Not surprisingly, the replication of FFV-BBtr, which contains only a truncated *bet* gene, is likewise strongly impaired in the presence of A3s [15,47,51].

FVs are retroviruses that differ from other viruses of this group in many aspects such as protein processing, morphogenesis, gene expression, and replication [49,52,53]. FVs have not yet been associated with any disease and are considered apathogenic [54]. This feature makes FVs potential vectors for gene delivery and vaccination [53]. The known human FV isolates are results of zoonotic transmissions of diverse simian FVs to humans [55]. With respect to virus-host coevolution, FV show a very strong coevolution with their host and related species [56,57]. At current, FV research focuses mostly towards all aspects of vector development, host-virus coevolution and the potential of interspecies transmission to other hosts, including humans [54].

In this study, we analyzed the functional interaction between FFV Bet and feA3 restriction factors as a model for the situation in humans and NHPs, since the feA3 repertoire is less complex than that of these species. Using bioinformatics and reverse genetics, we identified conserved motifs in Bet and tested their importance. We identified the *bel2* sequence of Bet as the essential determinant for A3 inactivation. Moreover, this study shows that nearly the entire FFV Bel2 domain is required for feA3 binding and inactivation, supporting the view that Bet inactivates feA3 by creating strong complexes with this restriction factor [44,45,47].

## Results

### Bet contains six conserved motifs

**Figure 1:**
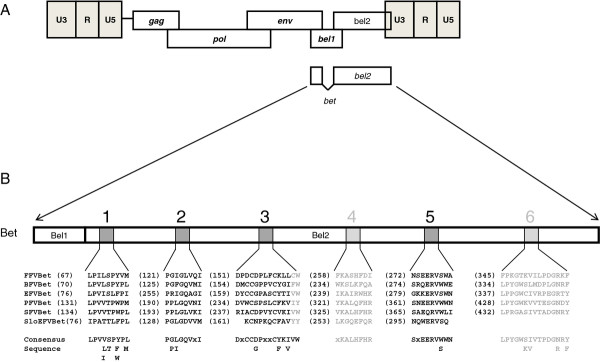
**Bet proteins of different FVs contain six conserved motifs. (A)** Genome organisation of FVs. The regulatory and accessory *bel1* and *bel2* genes are localized between *env* and the 3′LTR. The Bet protein is a product of a spliced transcript and consists of Bel1 and Bel2 parts. **(B)** Bioinformatics using the MEME program identified six conserved motifs localized in Bel2. Motifs are represented as gray boxes in the figure and the sequences are given below as well as the consensus sequence. Numbers in brackets indicate the position of the first amino acid of the motifs. In EFV Bet motifs 2 and 3 are found in reversed order. Motifs and residues that were not experimentally studied due to low degrees of conservation, such as motifs 4 and 6 and residues flanking motif 3, are represented in light shading and font, respectively. FFV, feline FV; BFV, bovine FV; EFV, equine FV; PFV, prototype/primate/human FV; SFV, simian FV; SloEFV, sloth endogenous FV.

All known FV Bet proteins consist of the N-terminus of Bel1 and the complete Bel2 sequence. However, sequence homology between Bet proteins of different FVs is very low [49]. To identify any possible conserved motifs amongst different Bet proteins, we performed bioinformatics using the MEME program, which searches for repeated amino acid patterns in given sequences [58]. Six conserved motifs, all localized within Bel2, were identified in the Bet proteins of bovine (BFV), equine (EFV), simian (SFV) FVs, FFV, PFV and SloEFV (Figure 1). MEME detected four motifs with standard settings (shown in black, motifs 1, 2, 3, 5); two additional motifs (4 and 6, marked in gray letters) were identified by searching with only the *bel2* sequences and using slightly modified parameters. The first five motifs are also conserved in the predicted Bet sequence of SloEFV, although the third and fifth motifs from SloEFV appear to be shorter than the corresponding motifs in other Bet proteins (Figure 1). In EFV Bet, the positions of the second and the third motifs are swapped with respect to the corresponding motifs in the other FV Bet proteins.

### The Bel2 part of FFV Bet is sufficient for feA3Z2b binding and inactivation

**Figure 2:**
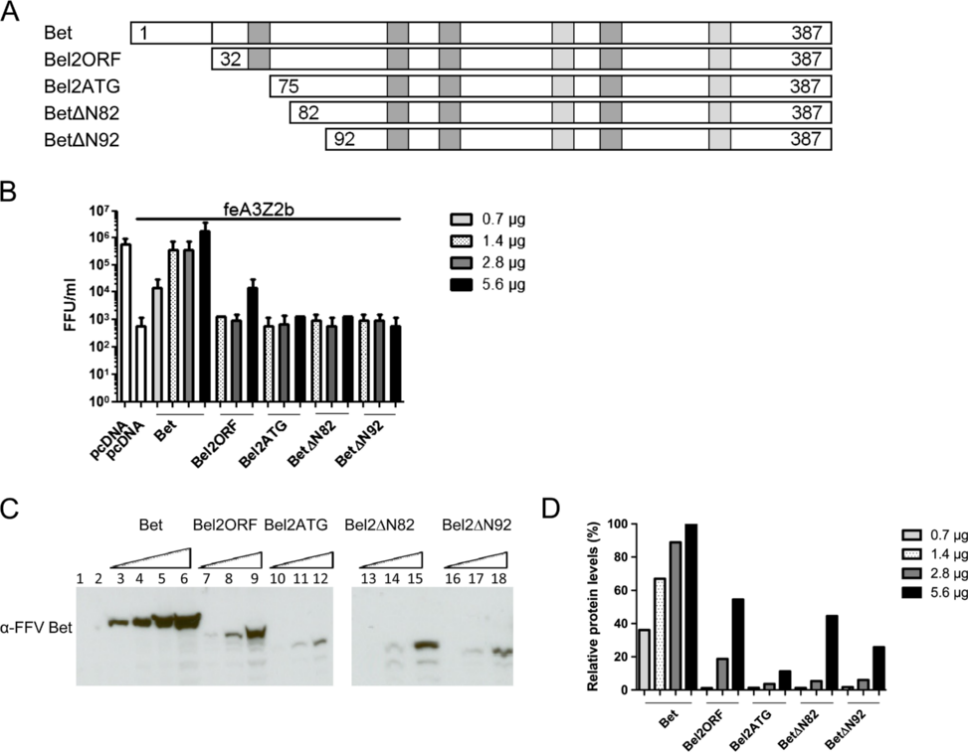
**Bet and Bel2ORF suppress feA3Z2b-mediated restriction. (A)** Schematic representation of full-length Bet and N-terminal Bet deletion mutants. Dark and light gray boxes represent the different MEME motifs in Bet. Bet and Bel2ORF contain all six conserved motifs, while downstream N-terminal Bet deletion mutants do not contain the first conserved motif. Numbers indicate the first and the last amino acids of the deletion mutants. **(B)** HEK293T cells were cotransfected with 4 μg of pCF-BBtr, 0.8 μg pcfeA3Z2b-HA, and increasing amounts of wt Bet and Bet deletion mutant expression plasmids as indicated in the legend. pcDNA was used to compensate for different plasmid amounts. Viral titers were determined in triplicate using the FeFab titration assay and are presented as mean values of three measurements; error bars represent standard deviations. Labels below the columns indicate cotransfected clones. The line above the columns indicates the presence of feA3Z2b. The first column shows the viral titer in the absence of feA3Z2b, the second, in the presence of feA3Z2b. The other columns show the titer in the presence of feA3Z2b and the coresponding Bet clones, as indicated in the figure. 0.7 μg of Bet expression plasmid and 5.6 μg of Bel2ORF expression plasmid yielded similar levels of feA3Z2b counteraction. In both cases, the titer increased more than 1 log. The other N-terminal deletion mutants did not counteract feA3Z2b-mediated restriction. **(C)** 40 μg of protein of transfected HEK293T cells was used for immunoblotting. Bet proteins were detected with the FFV Bet- specific serum. Proper loading was confirmed by detecting β-actin (data not shown). **(D)** Densitometric analysis of the relative levels of Bet protein expression. Wt Bet at 5.6 μg of transfected DNA was set to 100%. The legend indicates the amount of expression plasmid used to obtain corresponding protein amounts.

It was previously shown that FFV Bet binds and inactivates diverse feA3 proteins [15,47]. To determine the minimal FFV Bet sequence required for these two functions, N-terminal Bet deletion mutants were constructed (Figure 2A). Eukaryotic expression clone Bel2ORF encompasses the whole *bel2* ORF, while Bel2ATG starts with the first start codon inside *bel2* which is not conserved among known FVs. Bel2ORF contains all six conserved motifs, while Bel2ATG and mutants BetΔN82 and BetΔN92 lack the first motif.

We tested the ability of the mutants to bind to and/or inactivate feA3Z2b, the feA3 with the highest restriction potential against Bet-deficient FFV (FFV-BBtr) [33,47,51]. HEK293T cells were cotransfected with pCF-BBtr, the empty vector pcDNA, or pcfeA3Z2b-HA, and increasing amounts of plasmids encoding either wt or mutant Bet (Figure 2B). Two days post transfection (d.p.t.), viral infectivity was determined by titration using FeFab cells. Figure 2B shows that feA3Z2b resulted in a 3-log decrease of viral titer. Wt Bet efficiently counteracted this restriction in a dose-dependent manner. The highest concentration of Bel2ORF partially increased viral titer in the presence of feA3Z2b, while shorter deletion mutants did not have any effect on the viral titer. The presence of Bet in cell lysates was confirmed by immunoblotting with an FFV Bet-specific serum (Figure 2C). Levels of Bet deletion mutants were lower relative to the level of wt Bet, indicating instability of the deletion mutants (Figure 2C and D). As inactivation of feA3Z2b by Bet is concentration-dependent, the lower potential of Bel2ORF to counteract feA3Z2b may be due to the lower steady state of deletion mutants. As shown in Figure 2 panels B and D, at 0.7 and 5.6 μg transfected plasmid encoding Bet or Bel2ORF, respectively, slightly increased amounts of Bel2ORF protein resulted in comparable restoration of infectivity of the *bet*-deficient FFV genome in the presence feA3Z2b. Both wt and truncated Bet were detected with similar efficacy by the Bet antiserum and the V5 tag antibody, shown by comparative immunoblotting (Additional file 1).

**Figure 3:**
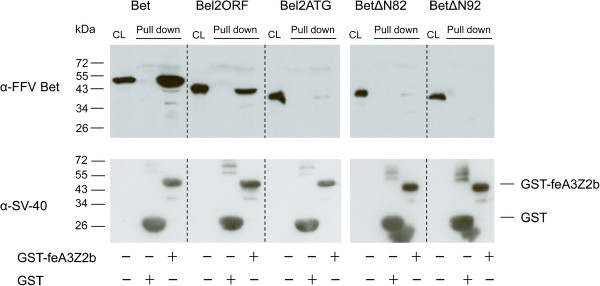
**Bet and Bel2ORF bind to feA3Z2b.** Bet and Bet N-terminal deletion mutants were expressed in 293T cells and pulled down either with GST or GST-feA3Z2b. Immunoblot analysis was performed with Bet-specific serum (upper panel) and an SV40 tag-specific antibody to detect the GST and GST-feA3Z2b fusion proteins (lower panel). Cell lysates (CL) of transfected cells were analyzed in parallel to confirm proper expression of wt and mutant Bet proteins. In addition to wt Bet, Bel2ORF was pulled down with GST-feA3Z2b. Due to high protein levels, some bands have ‘shades’ (lower panel). Symbols below the image indicate the presence (+) or absence (-) of GST or GST-feA3.

The ability of the truncated Bet proteins to bind feA3Z2b was tested in pulldown assays with bacterially expressed glutathione-S-transferase (GST) or GST-tagged feA3Z2b and lysates from HEK293T cells expressing wt or mutant Bet, as described previously [47]. Levels of N-terminal Bet deletion mutants were increased by supplementing the cell culture medium with 8 mM sodium butyrate. Bound proteins were precipitated using glutathione-coupled beads and detected by immunoblotting. In addition to wt Bet, known to bind to feA3Z2b [15,47], Bel2ORF was efficiently precipitated with feA3Z2b (Figure 3). Although low intensity bands are present in the case of Bel2ATG and BetΔN82, they were not reproducible and therefore considered to be unspecific. To confirm proper setup of the assays, the presence of similar amounts of GST and GST-feA3Z2b was confirmed with an SV40 tag-specific antibody (Figure 3 lower panel).

### C-terminal Bet deletion mutants do not counteract feA3Z2b-mediated restriction

To determine the minimal Bet sequence required for A3 inactivation, C-terminal deletion mutants were constructed (Additional file 2A). In transfected cells, the engineered proteins were present at only very low levels and thus, functional studies were conducted in the presence and absence of the proteasome inhibitor ALLN (Additional file 2B). HEK293T cells were transfected with pCF-BBtr, feA3Z2b, and wt or mutant Bet constructs. Cultures were treated for approximately 24 h with 25 μM of the proteasome inhibitor ALLN and tested for their ability to counteract feA3Z2b restriction. Both V5-tagged and untagged full-length Bet but none of the C-terminal Bet deletion mutants were able to inactivate feA3Z2b in the presence of ALLN or DMSO (solvent control) (Additional file 2B). The levels of wt Bet and wt Bet-V5 proteins did not change in the presence of ALLN, while the level of C-terminal deletion mutants increased due to proteasome inhibition (Additional file 2C).

The feA3Z2b binding ability of the C-terminal deletion mutants was also tested in pulldown assays. None of the C-terminal deletions bound to feA3Z2b, despite increased expression levels in transfected cells due to sodium butyrate addition (Additional file 2D).

### PFV Bet does not counteract or bind to feline A3Z2b

**Figure 4:**
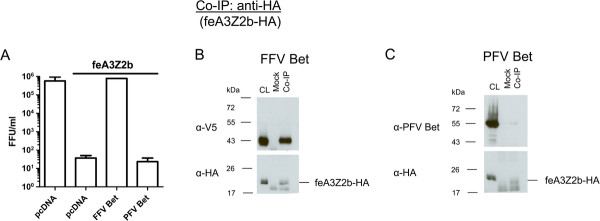
**PFV Bet does not counteract feA3Z2b. (A)** HEK293T cells were cotransfected with 4 μg of pCF-BBtr, 0.8 μg pcDNA or pfeA3Z2b, and 6 μg of plasmid expressing wt FFV or PFV Bet. Viral titers were determined two d.p.t. In the presence of feA3Z2b, FFV-BBtr titers decreased about 3 logs. Unlike FFV Bet, which completely rescues the viral titer, PFV Bet did not counteract feA3Z2b activity. The line above the graph indicates the presence of feA3Z2b. For co-IP experiments, 293T cells were cotransfected with 6 μg of plasmid expressing feA3Z2b-HA and 12 μg of FFV Bet **(B)** or PFV Bet **(C)**. Two d.p.t., cell lysates (CL) were subjected to co-IP with monoclonal mouse anti-HA IgG or beads only (mock). Precipitated FFV Bet but not PFV Bet proteins were detected by immunoblotting using monoclonal V5 tag-specific antibody and polyclonal hyperimmune sera against PFV Bet, respectively. feA3Z2b-HA, along with a lower molecular weight band that also appeared in mock samples, was detected in all samples using anti-HA IgG.

To circumvent the problems of the low stability of truncated FFV Bet proteins, we constructed chimeric FFV-PFV proteins. Bet proteins from different FVs are known to counteract A3s from cognate species but not from distantly related species. For instance, it has previously been shown that FFV Bet does not bind to human A3s [15] and that PFV Bet does not inactivate murine A3 [44,45]. We therefore first determined whether PFV Bet binds or inactivates feA3Z2b. HEK293T cells were cotransfected with pCF-BBtr, pcfeA3Z2b-HA or pcDNA, and plasmids expressing FFV or PFV Bet. As shown in Figure 4A, viral titers determined two d.p.i. decreased in the presence of feA3Z2b and were completely rescued by FFV Bet while, as anticipated, PFV Bet did not show anti-feA3 activity.

Since PFV Bet bound to glutathione beads incubated with GST (data not shown), the pulldown assay described above was not suitable for studying PFV Bet–feA3Z2b interactions, we thus used coimmunoprecipitation (co-IP) assays instead. HEK293T cells were cotransfected with pcfeA3Z2b-HA and plasmids expressing PFV or FFV Bet. Two d.p.t., co-IP was performed with monoclonal anti-HA IgG. Precipitated proteins were detected by immunoblotting. Unlike FFV Bet, PFV Bet did not coimmunoprecipitate with feA3Z2b-HA (Figure 4B and C). Immunoblotting with anti-HA IgG showed the presence of feA3Z2b-HA and an unspecific band of lower molecular mass (Figure 4B and C, lower panel). Incubation of cell lysates with beads only (without antibody, mock co-IP) did not result in unspecific feA3Z2b-HA precipitation.

### Chimeric FFV-PFV Bet proteins containing almost the entire FFV Bel2ORF bind and inactivate feA3Z2b

**Figure 5:**
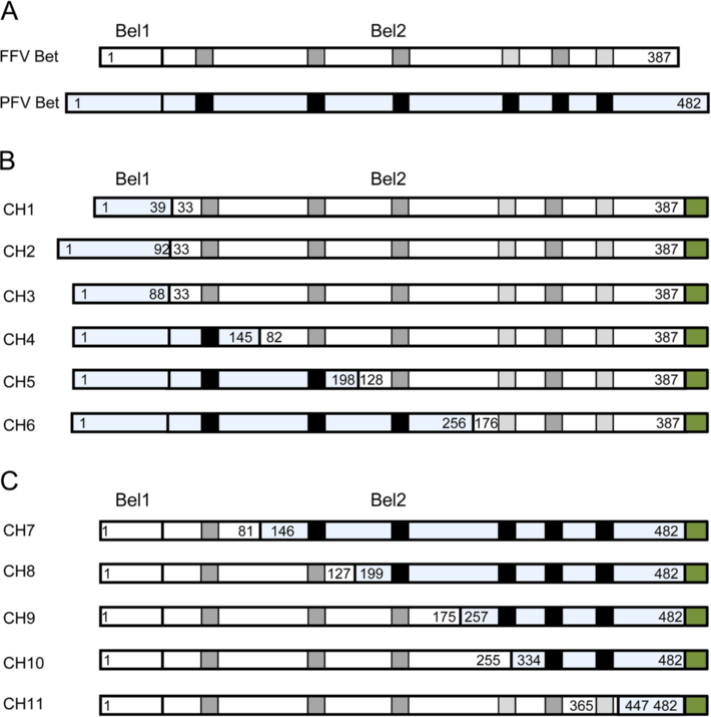
**Chimeric FFV/PFV Bet proteins. (A)** Schematic structure of FFV Bet and PFV Bet (blue shading). Conserved motifs are represented as gray boxes for FFV Bet and black boxes for PFV Bet. The Bel1part consists of 31 amino acids in FFV Bet and 88 amino acids in PFV Bet. FFV Bet contains 387 amino acids and PFV Bet, 482 amino acids. **(B)** FFV-PFV CH1 to CH6 are chimeras of N-terminal parts of PFV Bet and central and C-terminal parts of FFV Bet. **(C)** FFV-PFV CH7 to 11 are chimeras of N-terminal parts of FFV Bet and central and C-terminal parts of PFV Bet. White shading represents FFV Bet-derived sequences and blue shading, PFV Bet. Numbers indicate positions of the first and the last amino acid derived from PFV and FFV Bet. For example, CH1 is composed of the first 39 residues of PFV Bet and residues 33–378 of FFV Bet. A green box at the end of each protein represents the V5 tag.

To stabilize FFV Bet deletion mutants, eleven chimeric FFV-PFV Bet proteins, equivalent to the Bet deletion mutants described above, were constructed. In these chimeric proteins, deleted parts of FFV Bet were substituted by the equivalent parts of PFV Bet (Figure 5).

Both PFV and FFV Bet consist of Bel1 and Bel2 regions (Figure 5A), though the sizes of Bel1 regions of FFV and PFV Bet differ greatly, between 31 and 88 amino acids. In order to mimic this situation, three chimeric proteins containing the full FFV Bel2ORF and different lengths of PFV Bel1 were cloned as described in Methods. FFV-PFV CH1 contains the first N-terminal 39 amino acids of PFV Bel1 fused to the FFV Bel2ORF and is still 8 amino acids larger than the FFV Bel1 part but maintains a predicted extended secondary structure in the PFV sequence (data not shown). FFV-PFV CH2 contains PFV Bel1 and four additional residues from PFV Bel2 to avoid deletion of a predicted alpha helix in PFV Bet (data not shown). FFV-PFV CH3 contains the entire PFV Bel1 part fused directly to FFV Bel2ORF. FFV-PFV CH4 to 6 contain shorter C-terminal FFV Bet and longer N-terminal PFV Bet segments, while chimeric proteins CH7 to 11 are equivalent to FFV Bet C-terminal deletion mutants (Figure 5C). All chimeric proteins were V5-tagged to facilitate proper protein detection (Figure 5B and C).

**Figure 6:**
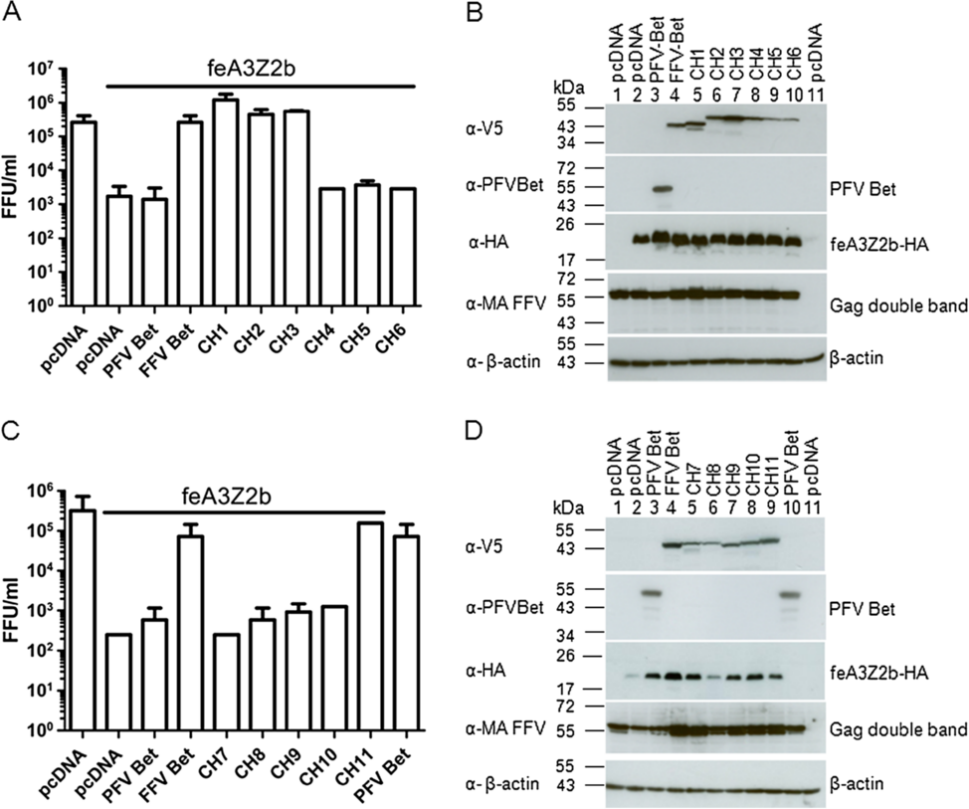
**The Bel1 domain and the C-terminal 22 amino acids of FFVBet can be replaced by PFV Bet sequences without loss of function.** HEK293T cells were cotransfected with 4 μg pCF-BBtr, 0.8 μg pcDNA or pfeA3Z2b, and 6 μg of plasmid expressing wt FFV Bet, PFV Bet or chimera, as indicated in the picture. **(A, C)** Two d.p.t., titration was performed and viral titers are represented as mean values of three independent experiments. Error bars represent standard deviations. In the presence of feA3Z2b, the FFV-BBtr titer decreased more than two logs. Unlike FFV Bet, which completely rescued titers, PFV Bet did not counteract feA3Z2b activity. Chimera CH1, 2, 3, and 11 completely restored the viral titer in the presence of feA3Z2b (indicated by the line above the graphs). **(B, D)** FFV Bet and chimeric Bet were detected with anti-V5 IgG while PFV Bet was detected with a PFV Bet-specific serum. Anti-HA IgG was used for feA3Z2b-HA detection, rabbit anti-matrix serum for Gag detection, and anti-β-actin IgG as a loading control.

To determine whether chimeric proteins CH1 to 6 inactivate feA3Z2b, HEK293T cells were cotransfected with pCF-BBtr, pcfeA3Z2b-HA, and plasmids encoding either wt PFV Bet, wt FFV Bet, or one of the chimeric FFV-PFV Bet CH1 to CH6 proteins. Viral titers determined 2 d.p.t. are shown in Figure 6A. As shown before, PFV Bet did not counteract feA3Z2b activity, while chimeric proteins CH1 to 3, containing the full-length FFV Bel2ORF and different PFV Bel1 sequences, fully restored FFV titers to levels similar to wt Bet (Figure 6A). Bet chimeras 4 to 6, with N-terminal FFV Bel2 sequences replaced by those from PFV, were non-functional and did not suppress feA3Z2b restriction.

FFV-PFV chimeric proteins CH7 to 11, carrying C-terminal PFV sequences of decreasing size were characterized as described above. Only FFV-PFV Bet CH11, carrying a short and obviously non-conserved C-terminal PFV Bet fragment, was functionally active against feA3Z2b.

The presence of proteins in cell lysates was confirmed by immunoblotting with the V5 antibody. Figure 6B and 6D show that the levels of chimeric proteins were comparable or slightly lower than those of wt FFV Bet. The PFV Bet antiserum efficiently detected full-length Bet but not chimera CH1 to CH5 and CH10 and CH11. In contrast, CH6 to CH9 were detectable only upon extended exposure (not shown).

**Figure 7:**
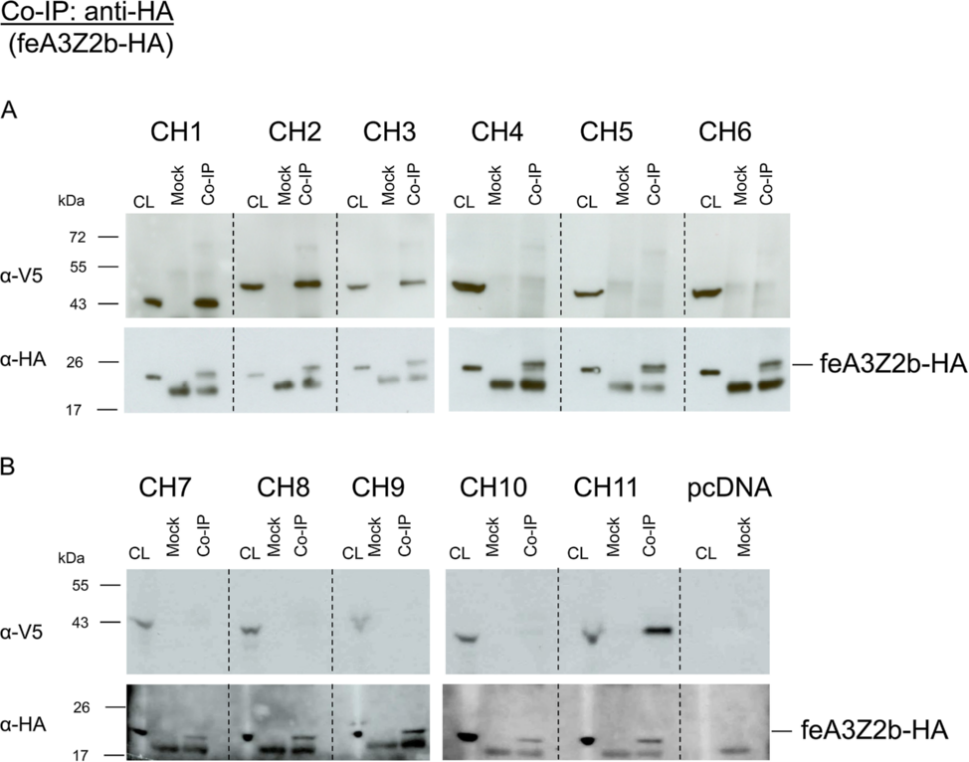
**FFV-PFV CH1, CH2, CH3 and CH11 bind to feA3Z2b-HA.** 293T cells were cotransfected with 6 μg of plasmid expressing feA3Z2b-HA and 12 μg of one of the chimeric protein expression plasmids (**A**, N-terminal chimera CH1 to CH6; **B**, C-terminal chimera CH7 to CH11) or pcDNA, as indicated in the picture. Two d.p.t., cell lysates (CL) were subjected to co-immunoprecipitation (co-IP) with monoclonal anti-HA IgG or beads only (mock). Precipitated proteins were detected by immunoblotting. feA3Z2b-HA was detected with an anti-HA monoclonal antibody in each sample, as expected. A lower molecular weight band that was also detected is considered unspecific, as it also appeared in mock samples. Hatched lines mark empty lanes used to separate individual experiments. Chimeric proteins were detected with anti-V5 IgG. In addition to FFV Bet, CH1, 2, 3, and 11 were coimmunoprecipitated by feA3Z2b.

Binding of chimeric FFV-PFV Bet proteins to feA3Z2b was studied by co-IPs as described above. As shown in Figure 7A and B, only FFV-PFV chimeric proteins CH1, 2, 3 and 11 coimmunoprecipitated with feA3Z2b-HA. Importantly, unspecific binding to the beads (mock co-IP) was not observed. Proper set up of the assay was confirmed by detection of feA3Z2b-HA (Figure 7, labelled α-HA in parts A and B).

### Identification of critical Bet residues by alanine scanning mutagenesis of conserved motifs

**Figure 8:**
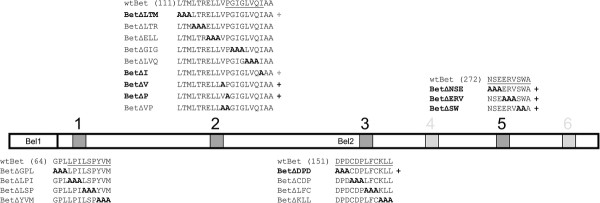
**Site-directed mutagenesis of the first, second, third and fifth conserved Bet motif.** FFV Bet contains 387 amino acids and consists of Bel1 and Bel2 sequences as indicated. Conserved motifs are represented as gray boxes and conserved motif 1, 2, 3 and 5 are underlined. Motifs 4 and 6 were not analyzed. Alanine residues that substitute the original residues are marked in bold. Numbers in brackets indicate the positions of the first amino acid in a given sequence. Mutations that did not impair Bet functions are marked with (+) and bolded names. Grey (+) signs indicates incomplete restoration of FFV titers by certain mutants.

To identify functionally important amino acids in the conserved Bet motifs, site-directed alanine scanning mutagenesis was performed. Amino acids in the conserved motifs or in flanking sequences were substituted by alanine as described in Methods and indicated in Figure 8, resulting in 20 FFV Bet mutants carrying one to three amino acid substitutions each. Although these Bet mutants are not classical deletion mutants, ∆ symbols were used to facilitate labelling of these substitutions.

The second conserved FFV Bet motif is localized directly behind the mutated part of FFV Bet-MCS, in which an MCS had been inserted inside *bel2*[51]. The MCS introduction resulted in the substitution of three and addition of four amino acids. Although the rest of Bet remained unchanged, Bet-MCS did not have anti-feA3 activity and did not bind to feA3 [15,47,51]. We therefore proceeded to more carefully characterize the second conserved motif together with these flanking sequences.

Functional feA3 inactivation and binding studies by GST-feA3Z2b pulldown were performed to determine whether mutant Bet proteins inactivate and/or bind feA3Z2b, as described above. The results are summarized in Figure 8 and provided in Additional files 3 and 4. As indicated in Figure 8, most mutations in or close to the first, second, or third motifs were detrimental to both Bet functions, while all 3 amino acid replacement mutants of the fifth motif displayed a wt phenotype with respect to feA3 inactivation and binding. Moreover, FFV Bet function was not impaired by single amino acid changes, while double amino acid mutations in motif 2 resulted in loss of function (Figure 8 and Additional file 3). In fact, BetΔV and BetΔP were both functionally active but the double mutant BetΔVP, in which both amino acids are replaced by alanine, was completely incapable of binding and counteracting feA3Z2b. As indicated in Figure 8 and shown in the Additional file 4, all mutants capable of functionally inactivating feA3Z2b also bound to this restriction factor in pulldown assays. In addition, BetΔLTM where mutations are outside of motif 2 and BetΔI where only the last amino acid of motif 2 were exchanged bound to feA3Z2b and partially inactivated this restriction factor. This attenuated phenotype was reproducibly detectable (data not shown). Moreover, BetΔGPL induced only a minor increase of the FFV titer (Additional file 3C) and it was not pulled down with GST-feA3Z2b (Additional file 4), which may indicate the low sensitivity of the pull down assay used. However, immunoblotting data show that protein expression levels of the Bet mutants were comparable to or slightly lower than wt Bet (Additional file 3D, E and F). Therefore, the lack of feA3Z2b counteraction by non-functional Bet mutants (in particular BetΔGPL) was not simply the consequence of low protein amounts.

In general, the binding properties of Bet substitution mutants were found to correlate well with their ability to inactivate feA3Z2b, although there were differences in the potential of individual mutants to inactivate feA3Z2b (Additional file 4).

### Mutant and wt Bet are localized in the cytoplasm and colocalize with feA3Z2b and feA3Z2a

**Figure 9:**
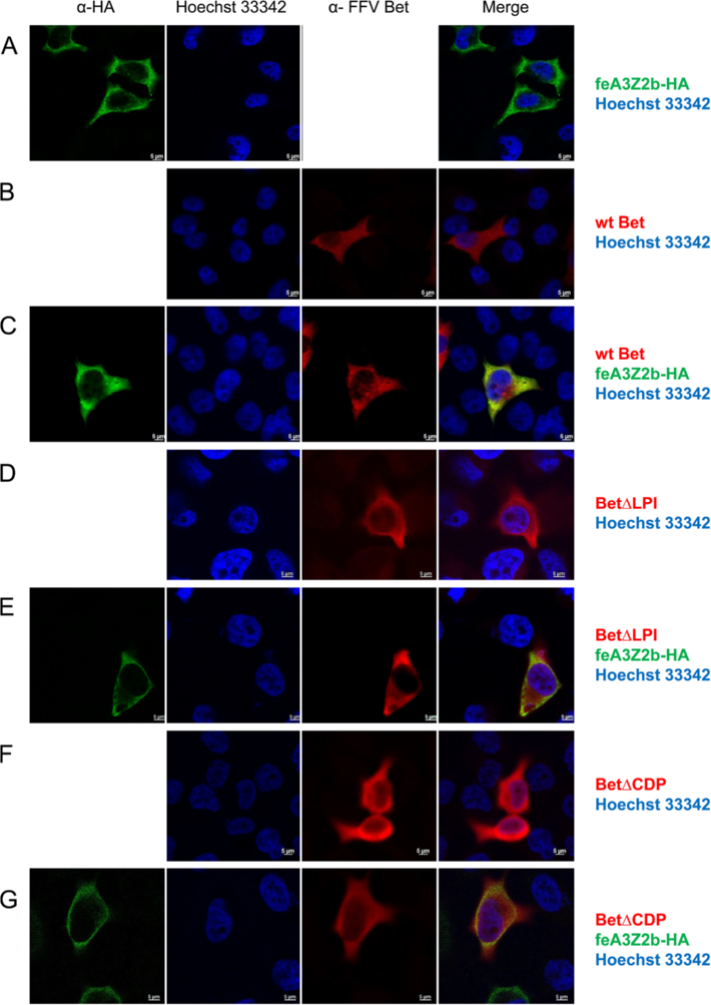
**Colocalization of feA3Z2b with wt and mutant Bet proteins.** HeLa cells were transfected with plasmids encoding HA-tagged feA3Z2b-HA **(A)** and/or Bet expression plasmids **(B – G)** as indicated on the right side of the image. feA3Z2b-HA was detected with HA tag-specific antibody (green), wt and mutated FFV Bet were detected by an FFV Bet-specific serum (red) and nuclei were stained with Hoechst 33342 (blue). The merge of feA3Z2b and Bet staining is shown in the right-hand column. feA3Z2b **(A, C, E, G)**, wt **(B, C)** and mutant Bet proteins **(D – G)** are predominantly localized in the cytoplasm. Inserted bars represent 5 μm.

Subcellular localization of mutant Bet proteins and their potential colocalization with feA3s were studied by indirect immunofluorescence (IIF) of paraformaldehyde-fixed HeLa cells using confocal microscopy. Bet was detected with an FFV Bel2-specific serum and is shown in red, while feA3Z2b was labeled with monoclonal anti-HA IgG and is shown in green (Figure 9). Both feA3Z2b-HA and wt Bet predominantly localize to the cytoplasm and, in cells coexpressing both proteins, there is strong colocalization throughout the cytoplasm without any indication of a Bet- or feA3Z2b-mediated relocalization of their corresponding binding partner. This (apparent) colocalization pattern indicates that the wt Bet–A3 complexes do not aggregate in specific regions of the cell but are rather evenly distributed throughout the cytoplasm. Nonfunctional mutant Bet proteins (Bet∆LPI, Bet∆CDP, FFV/PFV Bet CH4) were, similarly to wt Bet, also evenly distributed in the cytoplasm. In cells coexpressing these nonfunctional Bet mutants and feA3, there are regions of strong colocalization. However, colocalization detected by confocal microscopy does not prove the interaction of the two proteins but indicates that the two proteins have the same spatial occupancy within the cytoplasm. Based on these data, we conclude that the lack of feA3 inactivation by Bet mutants is not the consequence of the physical distance between both proteins.

**Figure 10:**
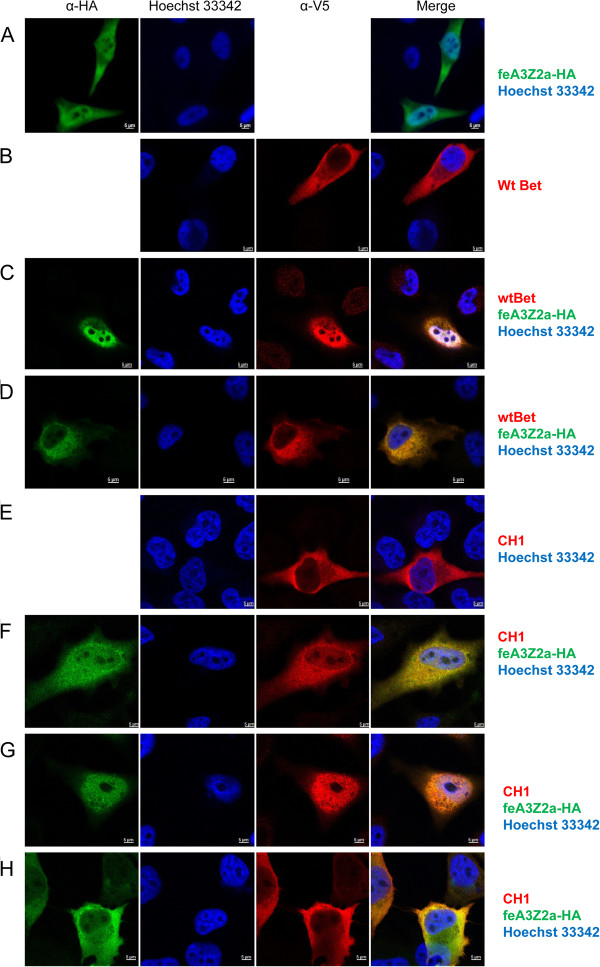
**Colocalization of feA3Z2a with functional Bet proteins.** HeLa cells were transfected with a plasmid encoding HA-tagged feA3Z2a-HA **(A)** and/or Bet expression plasmids (wt Bet or FFV-PFV Bet CH1, **B – H**), as indicated on the right. feA3Z2a-HA was detected with HA tag-specific antibody (green), FFV Bet was detected by an anti-V5 tag antibody (red) and nuclei were stained with Hoechst 33342 (blue). The merge of feA3Z2a and Bet staining is shown in the right-hand column. Wt Bet and FFV-PFV CH1 are localized in the cytoplasm **(B, E)** while feA3Z2a is localized both in the cytoplasm and the nucleus **(A)**. In some cells coexpressing feA3Z2a and functional Bet proteins, wt Bet and CH1 relocalize to the nucleus **(C, F, G)**. In other cells, feA3Z2a is recruited to the cytoplasm **(D, H)**. Inserted bars represent 5 μm.

**Figure 11:**
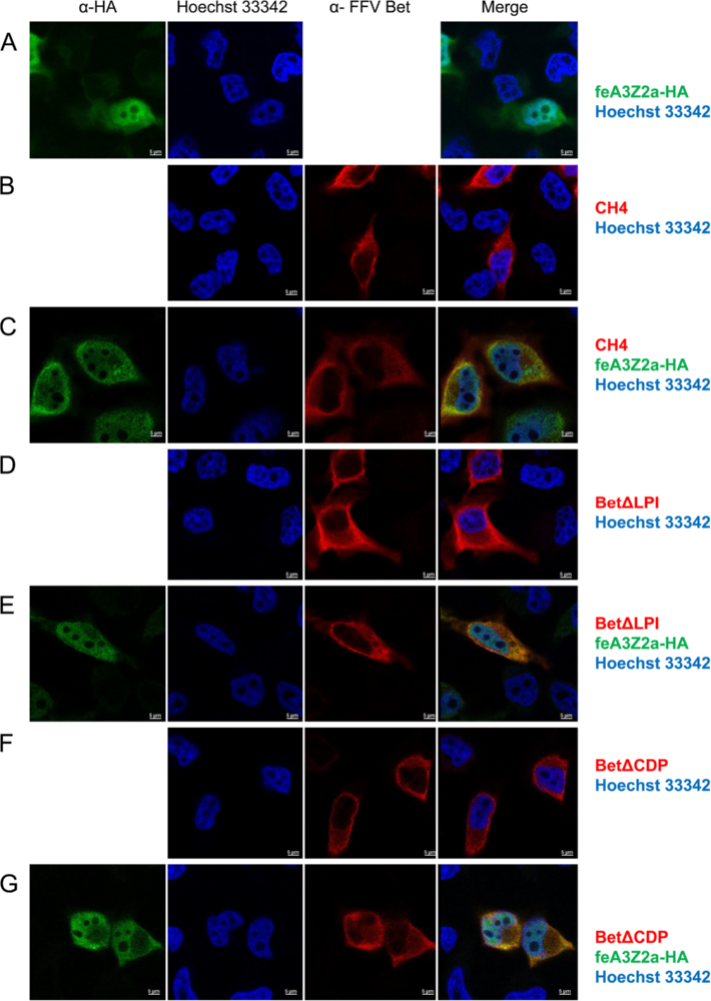
**Colocalization of feA3Z2a with nonfunctional Bet proteins.** HeLa cells were transfected with a plasmid encoding HA-tagged feA3Z2a-HA **(A)** and/or Bet expression plasmids **(B – F)**, as indicated on the right. feA3Z2a-HA was detected with HA tag-specific antibody (green), Bet proteins were detected by an FFV Bet-specific serum (red) and nuclei were stained with Hoechst 33342 (blue). The merge of the feA3Z2a and Bet staining is shown in the right-hand column. Nonfunctional Bet proteins are localized in the cytoplasm **(B, D, F)** while feA3Z2a is localized both in the cytoplasm and the nucleus **(A)**. In cells coexpressing feA3Z2a and a nonfunctional Bet mutant, there is no relocalization of proteins **(C, E, G)**. Inserted bars represent 5 μm.

Since feA3Z2a had been shown in independent studies to localize to the nucleus and the cytoplasm, we analyzed the colocalization of this feA3 isoform with wt Bet and some of the Bet mutants (Figures 10 and 11). In cells transfected only with V5-tagged wt Bet, Bet is predominantly found in the cytoplasm (Figure 10B), while in cells transfected with feA3Z2a, feA3Z2a localizes in both the nucleus and in the cytoplasm (Figure 10A). In most cells that express both wt Bet and feA3Z2a, these two proteins consistently colocalize in both compartments, meaning that Bet is recruited to the nucleus by feA3Z2a (Figure 10C). In addition, in some cells expressing both proteins, feA3Z2a and wt Bet predominantly localized in the cytoplasm (Figure 10D). Functional FFV-PFV Bet CH1 showed similar colocalization with feA3Z2a as wt Bet (Figure 10E-H). However, the non-functional Bet mutants tested were not recruited to the nucleus by feA3Z2a (Figure 11).

## Discussion

A3 proteins are cellular restriction factors that deaminate cytidine residues in ssDNA [8,19,34]. Two retroviral proteins that counteract A3 restriction factors have been described so far in detail: lentiviral Vif and FV Bet [8,15,44]. These two proteins counteract the A3 activity in different ways. While Vif induces proteasomal degradation of A3s [40,59-62], FV Bet does not change the steady state level of A3s [15,44,47]. It is assumed that Bet counteracts the activity of A3s by forming stable complexes with A3s, thus preventing them from being incorporated into virions [15,45,47].

Our results show that the Bel2 part of FFV Bet protein is essential for species-specific feA3Z2b inactivation and that this domain contains the feA3Z2b interaction site. In contrast, the Bel1 domain increases protein stability but it is not essential for the A3-inactivating function of Bet and can be exchanged between PFV and FFV Bet proteins without detectable loss of function. Alternatively, *bel1* RNA sequences or the splice junction may stabilize the transcripts or induce enhanced cytosolic protein biosynthesis. There is no sequence homology between PFV and FFV Bel1 (data not shown), leading to the question of whether the presence of any protein domain appended to the N-terminus of Bel2ORF would lead to the same increase of protein stability. However, since higher amounts of Bel2ORF than wt Bet are needed for the same level of A3 counteraction (Figure 2), it might be that the Bel1 domain contributes to anti-A3 activity, for instance, by interacting with some cellular effector protein.

In line with functional and binding studies, bioinformatics showed that the Bel2 parts of all FVs contain conserved motifs. Without knowing the protein structure of Bet, it is difficult to evaluate whether these motifs are localized on the protein surface and may thus directly comprise the feA3 binding site. By molecular modelling using the Robetta full-chain protein structure prediction server [63,64], we obtained hypothetical three-dimensional models of Bet. In the most probable model according to QMEAN and ProQservers [65,66], conserved motifs 1, 2 and 3, which have been shown here to be absolutely essential for Bet function, are localized on one side of the Bet protein surface (data not shown). Provided that this model reflects the real Bet structure, the feA3Z2b binding domain may consist of a combination of these motifs. Experimental structure determination of Bet, for instance by crystallography, is required to solve these issues. For instance, it is currently not known whether EFV Bet has a similar structure to the other Bet proteins, since the positions of the second and third conserved motifs are inverted relative to the other known FVs Bet proteins.

Considering the strong coevolution of FVs with their hosts including Bet and A3 proteins [2,56], the binding sites to the species-specific A3 forms may not be conserved between Bet proteins of different FVs. Alternatively, one can imagine that the A3 binding sites may be under highly dynamic, positive evolution. Thus, the A3 binding site may be highly divergent and their primary sequence/structure is likely not maintained. Following this idea, conserved motifs of Bet may be more important for maintaining the tertiary structure of Bet and for proper presentation of the species-specific binding site. The results of this study support both models, since most mutations in these motifs and in flanking sequences impaired the function of FFV Bet.

There is no start codon that would enable expression of the full-length motif 1 to 6-containing Bel2 protein and there is no conserved ATG start coding in the 5′ end of *bel2*. The first ATG in FFV *bel2* is, for instance, already downstream of the first essential motif, and such a Bel2-only protein (Bel2ATG) does not have the capacity to protect against A3 editing, as shown here. Since the *bet* splice acceptor is located at the 5′end of the *bel2* ORF of all FVs examined [49], Bet expression via a spliced *bel1-bel2* fusion transcript allows expression of all conserved *bel2* motifs. This mode of expression may be an efficient way to generate high amounts of Bet, since the internal promoter (IP) is very active upon full transactivation [49]. In addition, Bel1/Tas and Bet expression via alternative splicing of a single transcription unit may be an important regulatory mechanism of FV replication. Through regulated splicing of IP transcripts, early expression of Bel1/Tas may initiate and maintain high levels of viral gene expression, while later expression of Bet protects the viral genome from A3 editing during and after particle formation and reverse transcription. Expression of Bet during the late phase of replication and progeny virus production may therefore be the result of FV evolution to avoid A3 incorporation into virus particles during replication in A3-positive cells. It may be even more important for FVs due to the fact that reverse transcription may already occur in the virus-producing cells [52].

For all tested mutants, binding to feA3 correlated with the inactivation of this restriction factor. The tight association between feA3 binding and inactivation by FFV Bet supports the hypothesis that Bet inactivates A3s by creating strong complexes [15,45]. This mechanism of A3 inactivation requires high amounts of Bet proteins and may be the reason for high expression levels of Bet in FV-infected cells and animals [48,67,68]. In line with this, results of this study show that, for efficient inactivation of A3s, the amount of Bet must be at a sufficiently high level; small to modest amounts of Bet do not or only partially counteract A3 restriction.

Hypothetically, binding of Bet to feA3Z2b could mask the Gag binding site on feA3Z2b, thus preventing its incorporation into viral particles. Results presented here indicate that almost the whole Bel2ORF is important for feA3Z2b binding and suggest that Bel2ORF may wrap around feA3Z2b and prevent its interaction with Gag. Alternatively, binding of Bet to A3s could block transport of A3 into viral particles or interfere with some cellular factor(s) [15] crucial for A3 packaging into viral particles.

Confocal microscopy indicates that both Bet and feA3Z2b are apparently uniformly distributed in the cytoplasm and thus do not allow detection of protein relocalization upon complex formation. In contrast, feA3Z2a, which also restricts FFV and is inhibited by Bet binding [33,47], is also present in the nucleus. Wt Bet and functional Bet mutants colocalize to the nucleus in the presence of this restriction factor. Surprisingly, in some cells, feA3Z2a seems to retarget Bet which is somewhat counterintuitive since the smaller protein dictates sub-cellular localization of its larger binding partner. Why not all cells show this phenotype remains to be determined.

Bet is a highly expressed viral protein and a diagnostic marker for FV infection [51,67]. All known FVs express Bet, strongly supporting the importance of this protein for efficient FV replication. Sequences corresponding to *bet/bel2*, as determined by localization and sequence homology, have also been identified in the SloEFV, active more than 100 million years ago [43], indicating that Bet is an ancient gene. In addition, a sequence corresponding to *bel2* is even present in coelacanth endogenous FV [50], though it contains only the first conserved motif (data not shown). This may be a result of the accumulation of mutations from the lack of selective pressure leading to truncations of the parental *bel2* ORF.

Although Bet and Vif counteract A3 restriction factors, their localization in viral genomes and their fundamentally different mechanisms of A3 counteraction suggest that these two proteins may have evolved independently from each other. The ancestral protein(s) of Vif and Bet may have been cellular A3 binding proteins with regulatory functions. In this model, there was no need for high level of *vif* expression in lentiviruses, since Vif acts as an adaptor for a catalytic degradation of A3 proteins. In contrast, the internal promoter (IP) of FVs provides high levels of Bet for efficient inactivation of A3s simply by binding. Alternatively, FVs might have developed an IP to increase the expression of Bet, since Bet does not recruit the cellular degradation machinery.

## Conclusions

The Bel2 domain of FFV Bet contains conserved motifs and is essential for inactivation of feA3s. Although the Bel1 part is not directly involved in binding and inactivation of these restriction factors, it is important for expression of full-length Bel2ORF and protein stability. The absolute correlation between binding and inactivation of feA3Z2b by Bet mutants suggests that Bet inactivates A3s simply by creating strong complexes. Such a strong feA3Z2b-Bet binding is most probably a result of a very long coevolution of these two proteins. Considering that sloth and coelacanth FVs contain sequences that correspond to *bet*[43,50] and its conserved motifs, it is possible that this coevolution between Bet and A3s or their progenitors extends far back in vertebrate evolution.

## Methods

### Cell culture and virological methods

HEK293T and FeFab cells were propagated as described before [69]. HEK293T cells were seeded in 6 cm or 10 cm dishes and transfected using a modified calcium phosphate method [69]. FFV titers were determined by a β-galactosidase assay using FFV-FAB (FeFab) cells grown in 96-well plates [69]. FFV-containing supernatants were serially diluted 1:5, titrations were done in triplicate.

### Plasmids and DNA transfection

pFeFV-BBtr and pcfeA3 plasmids have been already described [33,51,69]. The bacterial expression vector for glutathione-S-transferase (GST)-tagged feA3Z2b has been described recently [47].

### Molecular cloning of FFV Bet mutants

To construct Bet N-terminal deletion mutants, the corresponding *bel2* sequences were amplified by Bel2ORF, Bel2ATG, FFVBetΔN82, or FFVBetΔN92 sense primers (containing a HindIII site) and FFVbel2as antisense primer using plasmid pBC-FFV-Bet as template [51]. All reactions were performed with Phusion™ High-Fidelity DNA Polymerase (NEB, Germany). Blunt-ended products were digested with HindIII and inserted into HindIII/SmaI-digested pBC12CMV.

To generate a V5-tagged Bet, *bet* was amplified with Bet sense and Bet-V5 antisense primers as above and cloned into HindIII/SmaI-digested pBC12CMV. To introduce V5 into Bet N-terminal deletion mutants, AccI/XmaI fragments of pBC-Bel2ORF, pBC-Bel2ATG, pBC-BetΔN82 and pBC-BetΔN92 were replaced by the corresponding fragment of pBC-FFVBetV5.

V5-tagged C-terminal Bet deletion mutants were constructed by amplification of corresponding *bet* sequences with Bet sense and one of three different antisense primers (dC1-V5, dC2-V5 or dC3-V5, Table 1) with a V5 epitope. PCR products were cloned into HindIII/SmaI-digested pBC12CMV.

**Table 1:** Primers used for cloning and site-directed mutagenesis

**Name**	**Sequence (5′-3′ direction)**
**Cloning of N-terminal deletion mutants of FFV Bet**	
FFVbel2ORF	ATCCC*AAGCTT*GCCACCATGGTCGGAAAGAATCCGGAAC (HindIII)
FFVbel2ATG	ATCCC*AAGCTT*GCCACCATGGCTTGGGACAACCCTC (HindIII)
FFVBet∆N82	ATATCCC*AAGCTT*GCCACCATGGTGGTCACACGTCTGGTG (HindIII)
FFVBet∆N92	ATATCCC*AAGCTT*GCCACCATGGAATCATGGAAGAAGTATC (HindIII)
FFVbel2as	*GGG*TCACTCGAGCTATTCAGAGTCAGATGACTC (SmaI)
**Cloning of V5 tagged FFV Bet and C-terminal deletion mutants of Bet**	
Bet-sense	CTCCCCTCG*AAGCTT*TCTGGGATATGTAAAACC (HindIII)
dC1-V5	*GGG*TCA**GGTGCTGTCCAGGCCCAGCAGGGGGTTGGGGATGGGCTTGCC**ATCATCAGCTTGTGCTCTCC (SmaI)
dC2-V5	*GGG*TCA**GGTGCTGTCCAGGCCCAGCAGGGGGTTGGGGATGGGCTTGCC**CAGCAGAGAGTATTCTCCTC (SmaI)
dC3-V5	*GGG*TCA**GGTGCTGTCCAGGCCCAGCAGGGGGTTGGGGATGGGCTTGCC**TCTTCCATCAGGAAGTATCAC (SmaI)
Bet-V5 antisense	*GGG*TCA**GGTGCTGTCCAGGCCCAGCAGGGGGTTGGGGATGGGCTTGCC**TTCAGAGTCAGATGACTCAG (SmaI)
**Alanine scanning mutgenesis**	
BetAlaRI	ATGCAAGATGAT*GGTACC*GCAGCGGCTCTAGTTAGCATAGTCAAATC (KpnI)
BetAlaRII	ATGCAAGATGAT*GGTACC*AACAATTCGGCAGCGGCCATAGTCAAATCCCTCTC (KpnI)
BetAlaRIII	ATGCAAGATGAT*GGTACC*ACAATTCTCTAGTTAGGGCAGCGGCATCCCTCTCCCCACAATC (KpnI)
BetAlaFI	ATGCAAGATGAT*GGTACC*AGCCGCTGCCCTGGTACAAATCGCCGCTAC (KpnI)
BetAlaFII	ATGCAAGATGAT*GGTACC*AGGAATAGGCGCCGCTGCCATCGCCGCTACACTTAC (KpnI)
BetAlaFIII	ATGCAAGATGAT*GGTACC*AGGAATAGGCCTGGTACAAGCCGCCGCTACACTTACTAAAACC (KpnI)
BetAlaR2	AGGTAAAAGATTCCTAT*GTCTACG*CACAATC (AccI)
R-XhoI	ATGCAAGATGAT*GGTACC*AACAATTCTCGAGTTAGCATAGTC (KpnI)
ForwardVP	5′ATGCAAGATGAT*CTCGAG*AATTGTTGGCTGCCGGAATAGGCCTGGTAC (XhoI)
ForwardV	5′ATGCAAGATGAT*CTCGAG*AATTGTTGGCCCCAGGAATAGGCCTGGTAC (XhoI)
ForwardP	ATGCAAGATGAT*CTCGAG*AATTGTTGGTAGCCGGAATAGGCCTGGTAC (XhoI)
dGPL-AS1	TGGAAGGGCAGCGGCGGGAACATCCTGCTTCTTG
dGPL-S2	GCCGCTGCCCTTCCAATTCTGAGTCCG
dLPI-AS1	ACTCAGGGCAGCGGCGAGTGGGCCGGGAACATCC
dLPI-S2	GCCGCTGCCCTGAGTCCGTATGTAATGG
dLSP-AS1	TACATAGGCAGCGGCAATTGGAAGGAGTGG
dLSP-S2	GCCGCTGCCTATGTAATGGCTTGGGACAACC
dYVM-AS1	CCAAGCGGCAGCGGCCGGACTCAGAATTGGAAG
dYVM-S2	GCCGCTGCCGCTTGGGACAACCCTCAG
dDPD-AS1	ATCACAGGCAGCGGCGGTTCTAGAACCTGTAATAC
dDPD-S2	GCCGCTGCCTGTGATCCTTTGTTCTGTAAG
dCDP-AS1	GAACAAGGCAGCGGCATCTGGGTCGGTTCTAGAAC
dCDP-S2	GCCGCTGCCTTGTTCTGTAAGTTGTTATGC
dLFC-AS1	CAACTTGGCAGCGGCAGGATCACAATCTGGGTCGGTTC
dLFC-S2	GCCGCTGCCAAGTTGTTATGCTGGAAAC
dKLL-AS1	CCAGCAGGCAGCGGCACAGAACAAAGGATCACAATC
dKLL-S2	GCCGCTGCCTGCTGGAAACAAAATATAC
dNSE-AS1	CCTCTCGGCAGCGGCCCCAGAGGCACTTCCAAATATG
dNSE-S2	GCCGCTGCCGAGAGGGTGTCATGGGCCAAAG
dERV-AS1	CCATGAGGCAGCGGCCTCACTGTTCCCAGAGGCAC
dERV-S2	GCCGCTGCCTCATGGGCCAAAGAGAATTC
dSW-AS1	CTCTTTGGCAGCGGCCACCCTCTCCTCACTGTTCC
dSW-S2	GCCGCTGCCAAAGAGAATTCTCACAGAG
AS2-XmaI	AGTGTAAGTTCA*CCCGGG*TCA*CTCGAG*CTATTCAGAGTCAGATGACTC (SmaI, XmaI)
**Cloning of chimeric FFV/PFV Bet fusion proteins**	
chimera-s1	ATGCAAGATGAT*AAGCTT*TAGCTGCAGCAACAAAG (HindIII)
ch1-as1	GTGTTCCGGATTCTTTCCAGCAATAGTCAGCTCTCC
ch1-s2	GGAGAGCTGACTATTGCTGGAAAGAATCCGGAACAC
ch2-as1	GTGTTCCGGATTCTTTCCCTTCTGAGCAATCATTTC
ch2-s2	GAAATGATTGCTCAGAAGGGAAAGAATCCGGAACAC
ch4-as1	CCACGTGTGACCACGTTTGCATAGTGATCCTGGCTC
ch4-s2	GAGCCAGGATCACTATGCAAACGTGGTCACACGTGG
ch5-as1	GTAAGTGTAGCGGCGATCTGAATGTTCACCTGACC
ch5-s2	GGTCAGGTGAACATTCAGATCGCCGCTACACTTAC
ch6-as1	CAGGCACCATTCTTCTAGTTGCTTTTGGCCCATTGC
ch6-s2	GCAATGGGCCAAAAGCAACTAGAAGAATGGTGCCTG
ch3-as1	GTGTTCCGGATTCTTTCCCATTTCCTCTGGTGTGGGGATCC
ch3-s2	GGATCCCCACACCAGAGGAAATGGGAAAGAATCCGGAACAC
ch-sense1- bspei	ATGCAAGATGA*TTCCGGA*ACACCCAAGACGGATC (BspEI)
ch7-as1	GATTCCAAAGAGGGTTGGCTGAGGGTTGTCCCAAGC
ch7-s2	GCTTGGGACAACCCTCAGCCAACCCTCTTTGGAATC
ch8-as1	CTGATAATTCTTATAAAATTGTACCAGGCCTATTCC
ch8-s2	GGAATAGGCCTGGTACAATTTTATAAGAATTATCAG
ch9-as1	GTCTGCACAGCCAGGTTTTTTGGTTACACTCTCTAGGGTC
ch9-s2	GACCCTAGAGAGTGTAACCAAAAAACCTGGCTGTGCAGAC
ch10-as1	AATCCTAGGATTGGTGAAGACTCTGGGATACAGGGAAGC
ch10-s2	GCTTCCCTGTATCCCAGAGTCTTCACCAATCCTAGGATT
ch11-as1	CTGTCAATGTTCTGATCTTCTCAGGATCACAGGCTATG
ch1-s2	CATAGCCTGTGATCCTGAGAAGATCAGAACATTGACAG
chimera-as	GGGTCA**GGTGCTGTCCAGGCCCAGCAGGGGGTTGGGGATGGGCTTGCC**GAAGGGTCCATCTGAGTC (SmaI)

All primer sequences are in the 5′ to 3′ orientation. Sequences in italics correspond to restriction enzyme recognition sites that were used for cloning; the names of the enzymes are given in brackets. Underlined sequences mark the mutations introduced. Sequences encoding the V5 tag are given in bold-face letters.

To construct chimeric Bet proteins, corresponding parts of PFV and FFV *bet* were fused in-frame by fusion PCR [70]. Corresponding parts of each gene were amplified in individual PCR reactions. For instance, for cloning of FFV-PFV CH1, PCR1 with chimera-s1 and ch1-as using pBC-FFV-Bet as template and PCR2 with ch1-s2 and Bet V5 as primers and pBC-PFV-Bet as template were performed as given above. Finally, the amplicons were fused in PCR3 using chimera-s1 and Bet-V5 antisense primers. Blunt-ended PCR products were digested with HindIII and cloned into HindIII/SmaI-digested pBC12CMV. To construct FFV-PFV CH7 to CH11, FFV Bet was amplified with generic ch-sense1-bspe1 and a mutant-specific as1 primer. PFV Bet was amplified with the corresponding s2 primer and generic chimera-as primer. Products of both PCRs were fused in a third PCR reaction with ch-sense1-bspeI and chimera-as primers. Final products were cloned into BspEI/SmaI digested pBC-Bet-V5.

Alanine scanning mutagenesis of the second conserved motif of FFV Bet and flanking residues was performed by amplifying *bet* between the unique HindIII and KpnI sites using Bet-sense and one of three antisense primers (BetAlaRI, BetAlaRII, BetAlaRIII for ΔELL, ΔLTR and ΔLTM, respectively). Products were cloned into HindIII/KpnI-digested pBC-Bet. Residues downstream of KpnI were mutated by amplifying *bet* from KpnI to AccI using sense primers (BetAlaFI, BetAlaFII and BetAlaFIII for ΔGIG, ΔLVQ and ΔI, respectively) and BetAlaR2 as general antisense primer. Products were cloned into pBC-Bet with KpnI and AccI. Using V, P, or VP sense primers, the BetAlaR antisense primer, and Bet-XhoI (in which an XhoI site was silently introduced with Bet sense and R-XhoI antisense primer) as template, corresponding parts of Bet were amplified and the indicated codons exchanged by alanine codons. PCR products were cloned into pBC-Bet-XhoI using XhoI and AccI.

Residues in the first, third and fifth conserved motifs were exchanged by fusion PCR. *Bet* was amplified with Bet-sense and AS1 antisense primers. The second PCR was performed with S2 sense primers and AS-XmaI primer, covering the *bet* stop codon and carrying an XmaI site (e. g. fusion PCR with Bet-sense, dGPL-AS1, dGPL-S2, AS-XmaI primers for ΔGPL). The two fragments were fused by PCR using Bet-sense and AS-XmaI. The final product was cloned into pBC12-CMV using HindIII and XmaI.

### Coimmunoprecipitation (co-IP) and immunoblotting

To study the interaction between proteins expressed in eukaryotic cells, HEK293T cells were seeded in 10 cm dishes and transfected with 6 μg of pcfeA3Z2b-HA and 12 μg of PFV Bet, FFV Bet, or chimeric FFV/PFV Bet expression plasmid. Two d.p.t., cells were lysed in TLB (20 mM Tris, pH 7.4, 137 mM NaCl, 10% glycerol, 2 mM EDTA, 1% Triton X-100 and protease inhibitor). Lysates were cleared by 5 min centrifugation at 500 × g. 100 μl of the cell lysate was incubated with monoclonal mouse anti-HA IgG (Abcam, Cambridge, UK) and protein-G-sepharose overnight at 4°C. The beads were washed three times in TLB. After the last wash, beads were boiled in sample buffer and precipitated proteins were detected by immunoblotting. feA3Z2b-HA was detected with monoclonal anti-HA IgG (Abcam). PFV Bet was detected with PFV Bet-specific serum. Wt FFV Bet and chimeric proteins were detected with monoclonal mouse anti-V5 IgG (Sigma-Aldrich, Munich, Germany). Membranes were incubated with horseradish peroxidase-conjugated secondary antibodies (Sigma-Aldrich) and visualized by enhanced chemiluminescence (ECL, GE Healthcare, Freiburg, Germany). Densitometry was performed of scanned autoradiograms using the ImageJ software [71].

### Immunofluorescence and confocal microscopy

HeLa cells were grown on coverslips in 6-well plates and transfected with 1 μg feA3Z2b and feA3Z2a expression plasmids and 2 μg of plasmid expressing wt or mutant Bet. 2 d.p.t., cells were fixed with 3% paraformaldehyde in PBS for 15 min. and permeabelized with 0.1% Triton-X-100 for 7 min. Cells were incubated with rabbit FFV Bet-specific serum (1:1000) and monoclonal mouse anti-HA IgG (Abcam) diluted in 3% BSA in PBS. Alexa Fluor^®^ 488 goat anti-mouse IgG and Alexa Fluor^®^ 594 goat anti-rabbit IgG (both from Invitrogen, Karlsruhe, Germany) were used as secondary antibodies in a dilution of 1:2000. Nuclei were stained with Hoechst 33342 (1:2000). Cells were imaged using a Zeiss LSM700 confocal microscope (Zeiss, Jena, Germany).

### Protein pulldown assays

For pull down assays of the feA3 with FFV-Bet, HEK293T cells were transfected with 10 μg of FFV Bet expression plasmids. Two d.p.t., cells were lysed in 250 μl TLB and lysates were cleared by centrifugation. 100 μl of cleared supernatants were incubated with approximately one mg GST-feA3Z2b fusion proteins purified by glutathione-S-sepharose affinity chromatography according to the manufacturer’s instruction (GE Healthcare, UK) as described previously [47]. Samples were incubated overnight at 4°C and washed three times with TLB. As negative control, samples were incubated with GST alone. Samples were boiled in sample loading buffer and separated and probed by SDS-PAGE and immunoblotting.

### Bioinformatics

Conserved motives in Bet were identified using MEME (Multiple Expectation maximization for Motif Elicitation [57]). MEME is an unsupervised learning algorithm for discovering motifs in sets of protein or DNA sequences that quantifies/predicts the chance of interchangeability of defined residues in related sequences [57].

DNA and protein sequence analysis were performed using Geneious and ClustalW2. Molecular models of FV Bet were obtained using the Robetta Protein Prediction Server (http://robetta.bakerlab.org) [63,64]. Predicted models were evaluated using the QMEAN and ProQ servers [65,66].

## Competing interests

The authors declare no conflict of interests.

## Authors’ contributions

DSL designed and performed experiments. DSL and ML wrote the manuscript. JL and AMR contributed to data evaluation and helped in writing of manuscript. AHW, MM and JD performed bioinformatics and structural modeling of Bet.CM provided unpublished data on feA3Z2a nuclear localization and provided reagents and controls. ML designed and supervised the study. All authors read and approved the final manuscript.

## Supplementary Material

Additional file 1 **N-terminal deletion mutants are detected to similar degrees by the Bet-specific serum and a V5 tag-specific antibody.** HEK293T cells were transfected with 5 μg of pBC-Bet-V5, pBC-Bel2ORF-V5, pBC-Bel2ATG-V5, pBC-Bet∆N82-V5 or pBc-Bet∆N92-V5 and harvested 2 d. p.t. 40 μg of proteins from each cell lysate was used for protein detection. Two SDS gels were used for immunoblotting and one membrane was incubated with the FFV Bet-specific serum **(A)** and the other with the V5-specific antibody **(B)**. The wt and mutant Bet proteins were detected with similar efficacy using both Bet-specific serum and V5 tag-specific antibody. Click here for file

Additional file 2 **C-terminal Bet deletion mutants do not counteract feA3Z2b-mediated restriction. (A)** Schematic presentation of full-length Bet and C-terminal Bet deletion mutants. Grey boxes represent conserved motifs and green boxes represent V5 tags. **(B)** HEK293T cells were cotransfected with 4 μg of pCF-BBtr and pcDNA or pfeA3Z2b-HA and 5 μg of plasmids expressing wt Bet or Bet C-terminal deletion mutants, as indicated. One d.p.t., ALLN (25 μM) or DMSO was added to the cells. Two d.p. t., titration was performed in triplicate and mean titer values are presented. Error bars represent standard deviations. Labels below the columns indicate the clone that was cotransfected. The line above the columns indicates the presence of feA3Z2b. Bet and Bet-V5 efficiently restored viral titer in the presence of ALLN. C-terminal Bet deletion mutants did not restore viral titer, although expression levels increased in the presence of ALLN. **(C)** 40 μg of proteins from each cell lysate were used for immunoblot analysis. Wt and mutant Bet were detected either with V5-specific antibody or an FFV Bet serum. Levels of C-terminal deletion mutants partially increased in the presence of ALLN, while levels of the other proteins were unchanged. HA tag-specific antibody was used for feA3Z2b-HA detection, an FFV MA serum for Gag detection, and detection of β-actin confirmed proper loading of the samples. **(D)** HEK293T cells were transfected with 10 μg of pBC-Bet∆C244-V5, pBC-Bet∆C292-V5, pBCBet∆C357-V5 or pBC-Bet-V5. Protein expression increased by supplementing cell culture medium with 8 mM sodium butyrate. Two d.p.t., cells were lysed and incubated with affinity-purified GST or GST-feA3Z2b. Pulled down proteins were detected by immunoblotting with V5-specific antibody. Hatched lines mark empty gel lanes to separate individual assays. Only full-length Bet-V5 was pulled down with GST-feA3Z2b. The presence (+) or the absence (-) of GST and GST-A3Z2b are indicated; CL, cell lysate. Click here for file

Additional file 3 **Site-directed mutagenesis of conserved FFV Bet motifs 1 to 3 impair Bet function.** HEK293T cells were cotransfected with pCF-BBtr and pcDNA or pfeA3Z2b and plasmid expressing wt or mutant FFV Bet proteins as indicated. **(A, B, C)** Two d.p.t., titration was performed in triplicate using FeFab cells and mean values are represented; error bars represent standard deviation. The line above the graph indicates the presence of feA3Z2b. Black dots on white bars indicate motif 1 mutants; hatched bars, motif 2 mutants; striped bars, motif 3 mutants; white dots on black bars, motif 5 mutants; black bars, wt Bet; white bars, pcDNA (see also Figure 8). In the presence of feA3Z2b, the FFV-BBtr titer decreased 3 to 4 logs. FFV Bet, used as a positive control restored the viral titer in all cases. None of the Bet proteins with mutations in the first conserved motif were functionally active, although there were minor variations of the titer. Bet∆DPD is the only mutant with substitutions in the third conserved motif that was functionally active. All mutants with substituted amino acids in the fifth conserved motif counteracted feA3Z2b-mediated FFV-BBtr restriction with slightly reduced efficacies **(D, E, and F)**. 40 μg of proteins from each cell lysate were used for immunoblot analysis. Wt and mutant Bet proteins were detected with FFV Bet-specific serum. HA tag-specific antibody was used for feA3Z2b-HA detection, MA serum for Gag detection, and β-actin as a loading control. Click here for file

Additional file 4 **Bet proteins with mutations in conserved motifs 1–3 cannot bind feA3Z2b.** HEK293T cells were transfected with 10 μg of wt or mutant Bet expression plasmids. Two d.p.t., cells were lysed and incubated with affinity-purified GST or GST-feA3Z2b. After overnight incubation, pulled down proteins were detected by immunoblotting with FFV Bet-specific serum. Hatched lines mark empty gel lanes to separate individual pulldown assays. The presence (+) or the absence (-) of GST and GST-A3Z2b are indicated. The upper panel shows pull down assays performed with Bet mutants carrying mutations in the second conserved motif. Pulldown assays with mutations in the first, third or fifth conserved motif of Bet are boxed. Click here for file
