# Supplementary figures and images for: Identification of the feline foamy virus Bet domain essential for APOBEC3 counteraction
Source: Retrovirology. 2013 Jul 24;10:76. doi: 10.1186/1742-4690-10-76 (PMC3751544; doi:10.1186/1742-4690-10-76)

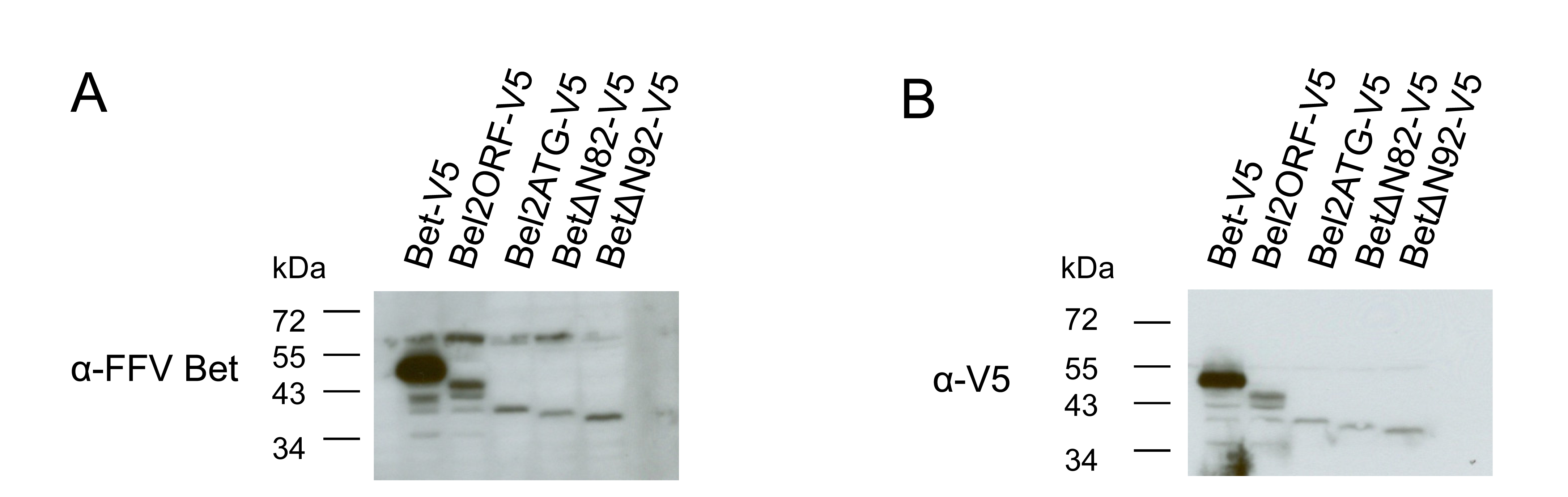

Supplement: Additional file 1 — N-terminal deletion mutants are detected to similar degrees by the Bet-specific serum and a V5 tag-specific antibody. HEK293T cells were transfected with 5 μg of pBC-Bet-V5, pBC-Bel2ORF-V5, pBC-Bel2ATG-V5, pBC-Bet∆N82-V5 or pBc-Bet∆N92-V5 and harvested 2 d. p.t. 40 μg of proteins from each cell lysate was used for protein detection. Two SDS gels were used for immunoblotting and one membrane was incubated with the FFV Bet-specific serum (A) and the other with the V5-specific antibody (B). The wt and mutant Bet proteins were detected with similar efficacy using both Bet-specific serum and V5 tag-specific antibody. [file 1742-4690-10-76-S1.tiff]

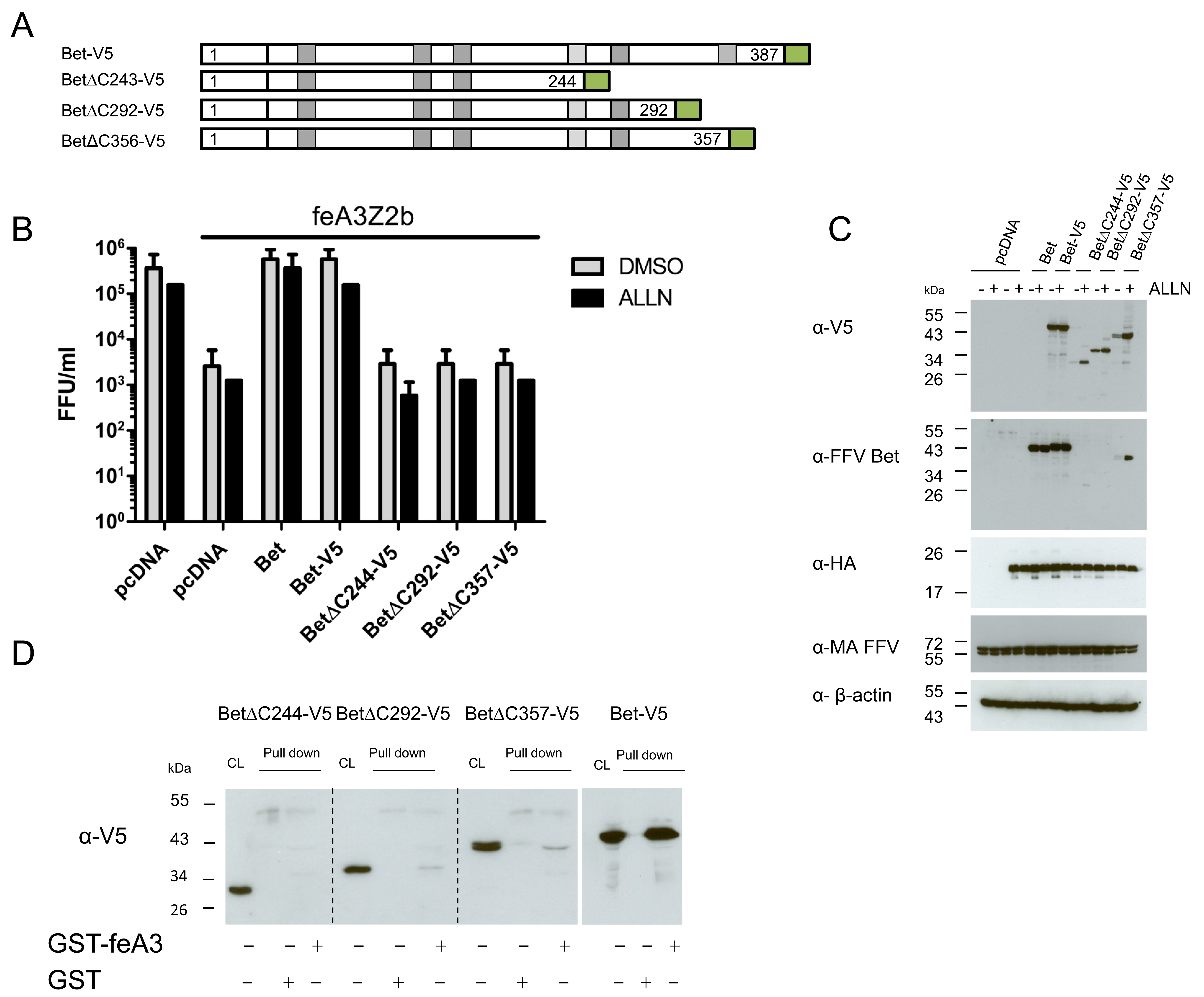

Supplement: Additional file 2 — C-terminal Bet deletion mutants do not counteract feA3Z2b-mediated restriction. (A) Schematic presentation of full-length Bet and C-terminal Bet deletion mutants. Grey boxes represent conserved motifs and green boxes represent V5 tags. (B) HEK293T cells were cotransfected with 4 μg of pCF-BBtr and pcDNA or pfeA3Z2b-HA and 5 μg of plasmids expressing wt Bet or Bet C-terminal deletion mutants, as indicated. One d.p.t., ALLN (25 μM) or DMSO was added to the cells. Two d.p. t., titration was performed in triplicate and mean titer values are presented. Error bars represent standard deviations. Labels below the columns indicate the clone that was cotransfected. The line above the columns indicates the presence of feA3Z2b. Bet and Bet-V5 efficiently restored viral titer in the presence of ALLN. C-terminal Bet deletion mutants did not restore viral titer, although expression levels increased in the presence of ALLN. (C) 40 μg of proteins from each cell lysate were used for immunoblot analysis. Wt and mutant Bet were detected either with V5-specific antibody or an FFV Bet serum. Levels of C-terminal deletion mutants partially increased in the presence of ALLN, while levels of the other proteins were unchanged. HA tag-specific antibody was used for feA3Z2b-HA detection, an FFV MA serum for Gag detection, and detection of β-actin confirmed proper loading of the samples. (D) HEK293T cells were transfected with 10 μg of pBC-Bet∆C244-V5, pBC-Bet∆C292-V5, pBCBet∆C357-V5 or pBC-Bet-V5. Protein expression increased by supplementing cell culture medium with 8 mM sodium butyrate. Two d.p.t., cells were lysed and incubated with affinity-purified GST or GST-feA3Z2b. Pulled down proteins were detected by immunoblotting with V5-specific antibody. Hatched lines mark empty gel lanes to separate individual assays. Only full-length Bet-V5 was pulled down with GST-feA3Z2b. The presence (+) or the absence (-) of GST and GST-A3Z2b are indicated; CL, cell lysate. [file 1742-4690-10-76-S2.tiff]

A

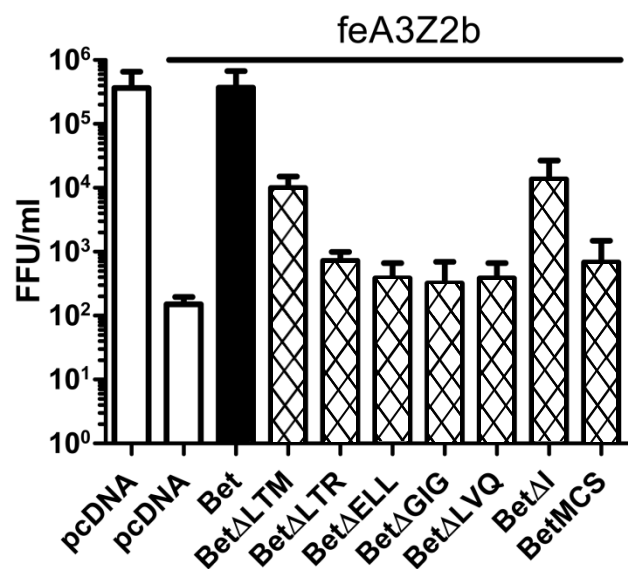

B

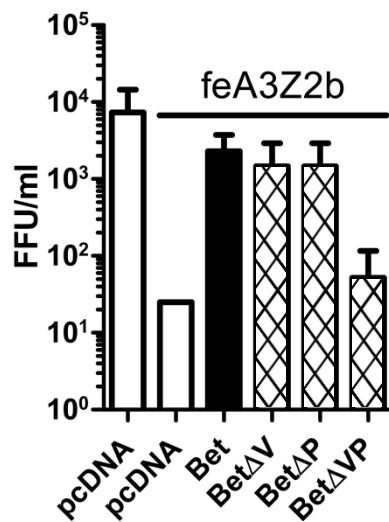

C

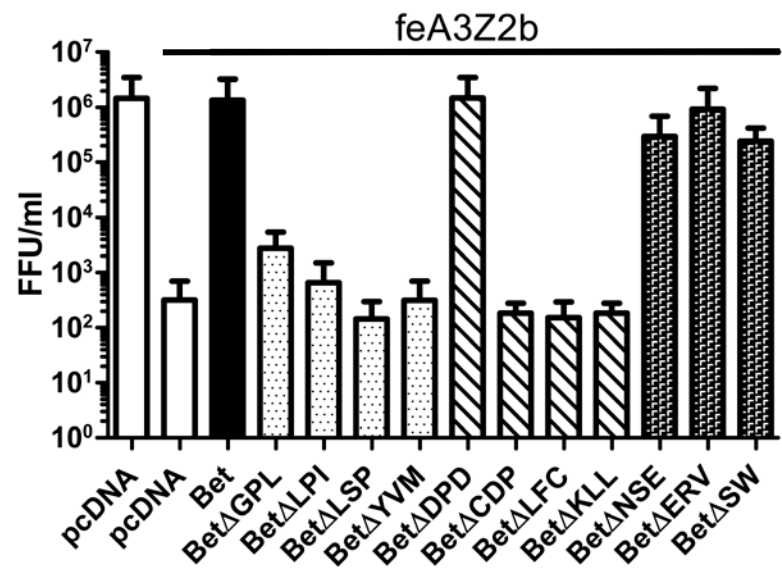

D

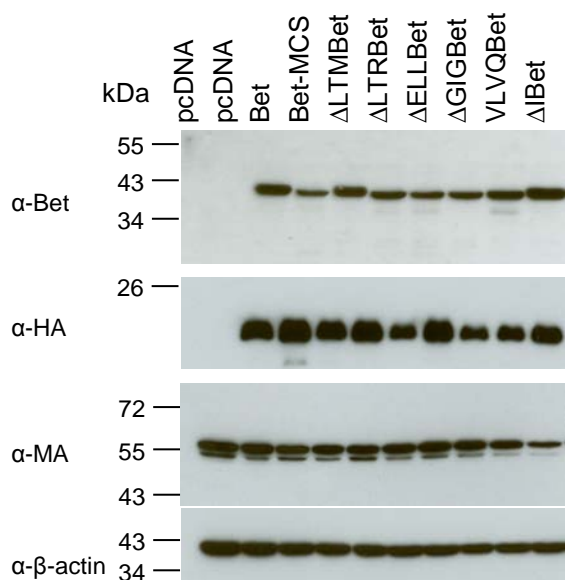

E

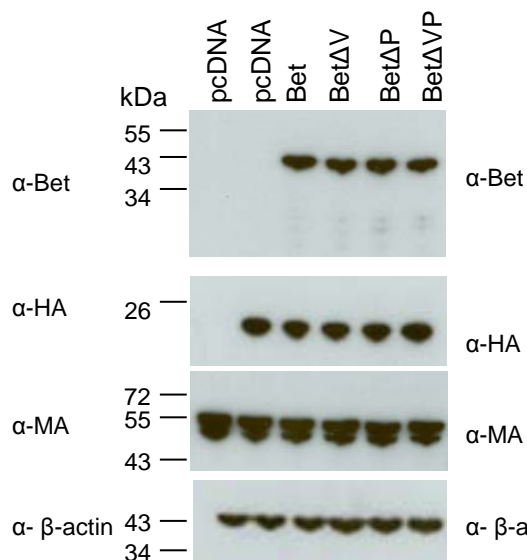

F

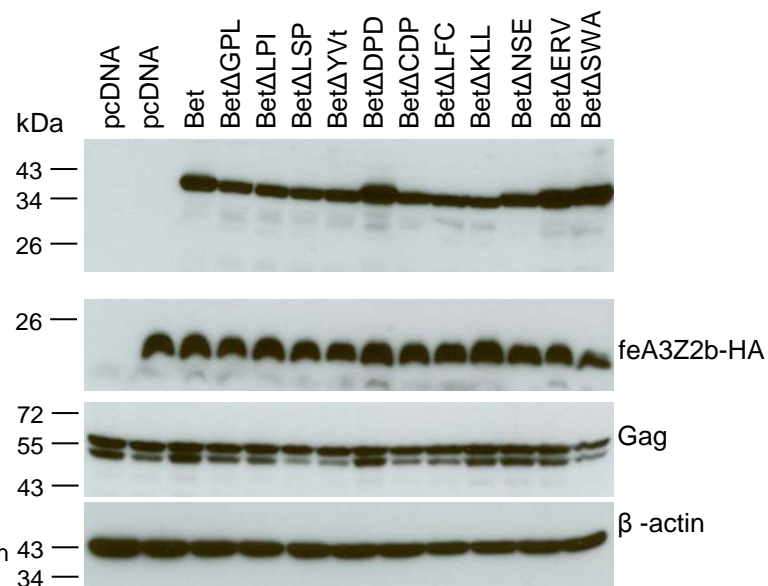

Supplement: Additional file 3 — Site-directed mutagenesis of conserved FFV Bet motifs 1 to 3 impair Bet function. HEK293T cells were cotransfected with pCF-BBtr and pcDNA or pfeA3Z2b and plasmid expressing wt or mutant FFV Bet proteins as indicated. (A, B, C) Two d.p.t., titration was performed in triplicate using FeFab cells and mean values are represented; error bars represent standard deviation. The line above the graph indicates the presence of feA3Z2b. Black dots on white bars indicate motif 1 mutants; hatched bars, motif 2 mutants; striped bars, motif 3 mutants; white dots on black bars, motif 5 mutants; black bars, wt Bet; white bars, pcDNA (see also Figure 8). In the presence of feA3Z2b, the FFV-BBtr titer decreased 3 to 4 logs. FFV Bet, used as a positive control restored the viral titer in all cases. None of the Bet proteins with mutations in the first conserved motif were functionally active, although there were minor variations of the titer. Bet∆DPD is the only mutant with substitutions in the third conserved motif that was functionally active. All mutants with substituted amino acids in the fifth conserved motif counteracted feA3Z2b-mediated FFV-BBtr restriction with slightly reduced efficacies (D, E, and F). 40 μg of proteins from each cell lysate were used for immunoblot analysis. Wt and mutant Bet proteins were detected with FFV Bet-specific serum. HA tag-specific antibody was used for feA3Z2b-HA detection, MA serum for Gag detection, and β-actin as a loading control. [file 1742-4690-10-76-S3.pdf]

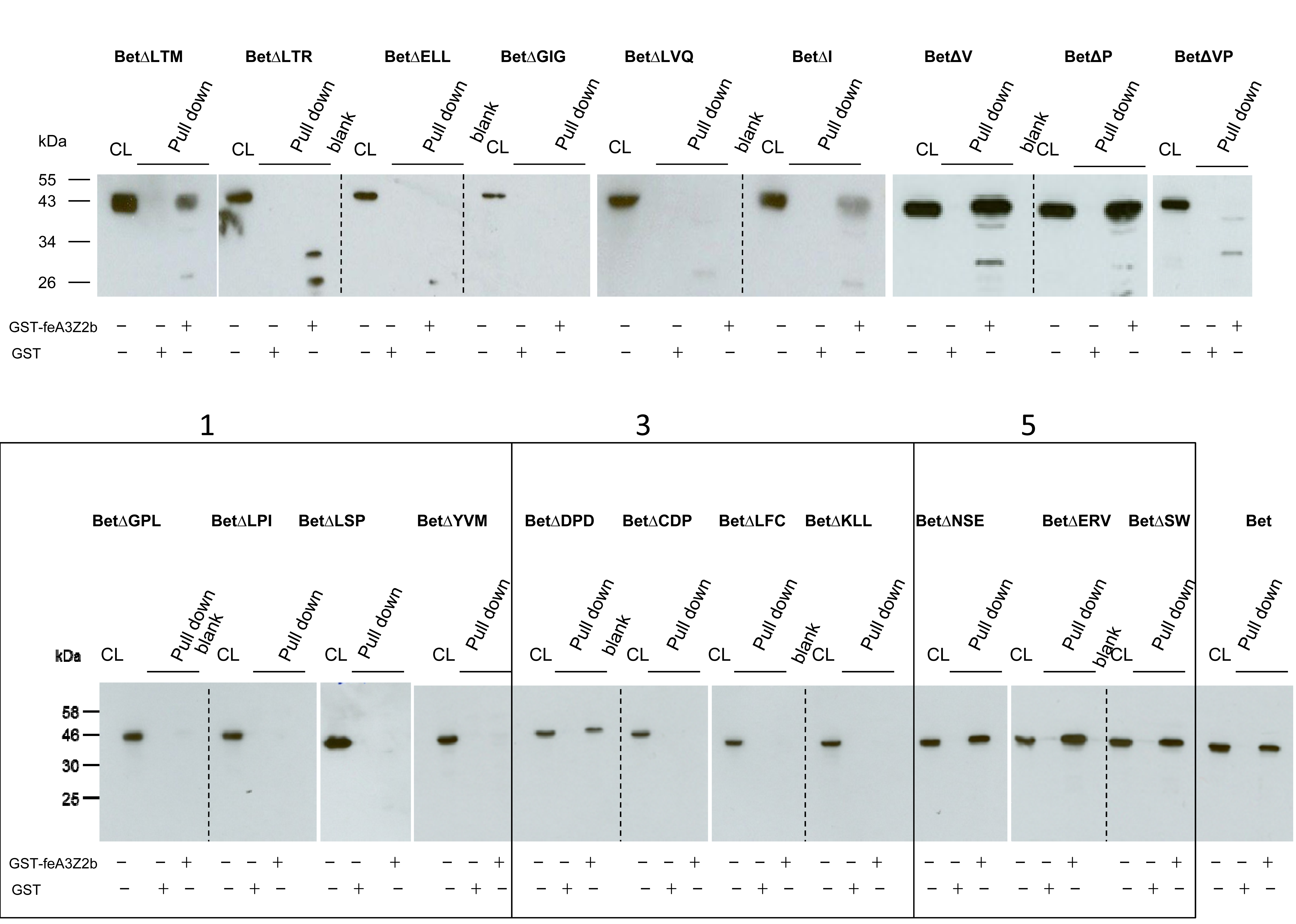

Supplement: Additional file 4 — Bet proteins with mutations in conserved motifs 1–3 cannot bind feA3Z2b. HEK293T cells were transfected with 10 μg of wt or mutant Bet expression plasmids. Two d.p.t., cells were lysed and incubated with affinity-purified GST or GST-feA3Z2b. After overnight incubation, pulled down proteins were detected by immunoblotting with FFV Bet-specific serum. Hatched lines mark empty gel lanes to separate individual pulldown assays. The presence (+) or the absence (-) of GST and GST-A3Z2b are indicated. The upper panel shows pull down assays performed with Bet mutants carrying mutations in the second conserved motif. Pulldown assays with mutations in the first, third or fifth conserved motif of Bet are boxed. [file 1742-4690-10-76-S4.tiff]
